# Identifying the clonal origin of synchronous multifocal tumors in the hepatobiliary and pancreatic system using multi-omic platforms

**DOI:** 10.18632/oncotarget.14018

**Published:** 2016-12-19

**Authors:** Weiqin Jiang, Yongfeng Ding, Yifei Shen, Longjiang Fan, Linfu Zhou, Zhi Li, Yi Zheng, Peng Zhao, Lulu Liu, Zhou Tong, Weijia Fang, Weilin Wang

**Affiliations:** ^1^ Cancer Biotherapy Center, First Affiliated Hospital, Zhejiang University, China; ^2^ Department of Surgical Oncology, First Affiliated Hospital, Zhejiang University, China; ^3^ Institute of Bioinformatics & Research Center for Air Pollution and Health, Zhejiang University, China; ^4^ Medical Biotechnology Laboratory, Zhejiang University, China; ^5^ Department of Radiology, First Affiliated Hospital, Zhejiang University, China; ^6^ Key Laboratory of Precision Diagnosis & Treatment for Hepatobiliary & Pancreatic Tumor, First Affiliated Hospital, Zhejiang University, China; ^7^ Division of Hepatobiliary and Pancreatic Surgery, Department of Surgery, First Affiliated Hospital, Zhejiang University, China; ^8^ Collaborative Innovation Center for Diagnosis and Treatment of Infectious Diseases, China

**Keywords:** synchronous multifocal tumors, hepatobiliary and pancreatic system, mutation, copy number variation, single clonal evolution

## Abstract

Synchronous multifocal tumors often pose a diagnostic challenge for oncologists. The purpose of this study was to determine the clonal origin and metastatic relationship of synchronous multifocal tumors in the hepatobiliary and pancreatic system using multi-omic platforms. DNA samples were extracted from three masses harvested from a 50-year-old Han Chinese male patient who suffered from synchronous multifocal tumors in the pancreatic tail, upper biliary duct, and omentum at the time of diagnosis. The clonal origin of these samples was tested using two platforms: next-generation sequencing (NGS) of 390 key genes harboring cancer-relevant actionable mutations and whole-genome copy number variation (CNV) chip analysis. The NGS approach revealed high mutational concordance, and the gene CNV profiles were similar between lesions. Whole-genome CNVs for the three samples were further investigated using an Affymetrix chip. Using matched CNV chip data from The Cancer Genome Atlas (TCGA), we developed a computational model that generated tissue-specific CNV signatures for hepatocellular carcinoma, pancreatic carcinoma, and cholangiocarcinoma to accurately identify the origin of the tumor samples. After adding the patient's CNV chip data to the model, all three samples were clustered into the pancreatic cancer branch. Both our NGS and CNV chip analyses suggested that clinically diagnosed synchronous pancreatic cancer and cholangiocarcinoma originated from the same cell population in the pancreas in our patient. This study highlights the use of genomic tools to infer the origin of synchronous multifocal tumors, which could help to improve the accuracy of cancer diagnosis.

## INTRODUCTION

Synchronous multifocal tumors across multiple tissues are common and mostly metastatic and sometimes include a small number of concurrent multiple primary tumors [1]. Determine their clonal origin is important since it can impact diagnoses, treatments, and follow-up management of patients [2]. Cancers in the hepatobiliary and pancreatic system exhibit similar anatomical and histological features, making identification of their clonal origin challenging. For example, it is difficult to distinguish primary cholangiocarcinoma and metastatic pancreatic adenocarcinoma in a liver biopsy.

Pathological diagnosis of primary tumors and metastatic deposits is usually determined through traditional analyses, such as histopathological and immunohistochemical approaches. However, these methods are prone to failure when the tumor status shifts from primary to metastatic [3, 4], when markers are shared within different primary cancers [5], or when other potential difficulties arise [6]. Among cancers, adenocarcinomas often lack markers that can efficiently trace the origin of the tumors, especially when cancer spreads to multiple organs [7]. Specific to the hepatobiliary and pancreatic system, many immunohistochemical markers have been tested to identify the primary site of a carcinoma of unknown primary site. However, most of these previously reported markers lack sensitivity, specificity, or positive likelihood ratio to warrant their clinical practice. Expression of cytokeratin (CK)7, 19, and 20 is often found in the immunohistochemical profiles of both pancreatic adenocarcinoma and cholangiocarcinomas [8]. N-cadherin has also been used as a marker, since it stains ~27% of the pancreas carcinomas and ~58% of the cholangiocarcinomas [9].

Cancer is known to be a “genomic disease” [10, 11]. The cancer cell population is characterized by a high incidence of somatic mutations, aberrant ploidies of chromosomes, and copy number variations (CNVs) [12]. Genomic sequencing has recently allowed inferring the clonality and metastasis of tumor masses [13, 14], particularly for cases that are unlikely to be identified using traditional approaches. For example, screening mutations of the consensus key cancer genes [15] (http://cancer.sanger.ac.uk/census) provides a means to examine not only the clonal evolution theory of tumor cells, but also their metastasis and origin [10, 16]. The Cancer Genome Atlas (TCGA) paves the way to characterize a more comprehensive landscape of oncogenic signatures across human cancers using whole-genome data. It is anticipated that TCGA will find clinical applications in the classification of cancers of unknown origin [17]. Indeed, performing sample-wise clustering in 12 different malignancies to derive subtypes based on 6 different data types from the TCGA showed that the patterns of copy number change varied across tissue type, and subtyping of the tumors based on CNVs revealed a significant correlation with tissue type [18]. Furthermore, using single-cell sequencing, CNVs can help to elucidate tumor evolution on an even finer scale [13]. Furthermore, the mechanism of metastasis for pancreatic adenocarcinoma could also be inferred using bulk DNA sequencing [19, 20].

Herein, we investigate whether “omic” platforms could be translated into clinical application, facilitating the identification of the clonal origin of synchronous multifocal tumors in the hepatobiliary and pancreatic system. Our proof-of-principle study demonstrates how genomic techniques at different omic levels can help to identify tumor origin and metastasis in patients whose cancers are characterized by synchronous multiple malignant tumors in the pancreatic tail, upper biliary duct, and omentum at the time of diagnosis.

## RESULTS

### Overview of somatic mutations

The average coverage of genes harboring somatic mutations was 575.8, 399.1, and 445.2 for the bile duct, omentum, and pancreas, respectively (Table 1). A total of 143 mutations were found in these three tissues. In the pancreas, 63 mutations were found in 40 genes (30 in exons), 84 somatic mutations were found in 52 genes (39 in exons) in the biliary duct, and 88 mutations were found in 59 genes (41 in exons) in the omentum. The detailed somatic mutation data for these three masses are listed in Supplementary Table S1. Within each tissue, the coverage and somatic mutation rates were both slightly higher in exons than in non-exons (Supplementary Figure S1). Of the detected mutations, 60.7%, 65.5%, and 76.2% had allele frequencies of less than 0.05 in the bile duct, omentum, and pancreas, respectively. As expected, low-frequency somatic mutations were only detected at very high coverage; therefore, the confidence in calling those low-frequency mutations should have been reasonably high. We found the same TP53 mutation (chr17 7577538 exon7 C = >T), with mutation frequencies of 0.156, 0.084, and 0.06 in the bile duct, omentum, and pancreas, respectively. The mutations that accumulate during pancreatic carcinogenesis are mainly focused on KRAS, TP53, CDKN2A and SMAD4 [21]. In consideration of tumor heterogeneity, we have not found KRAS, CDKN2A or SMAD4 mutations in our samples. Intriguingly, although KRAS mutations are reported for 99% of PanIN-1s37, no more than 95% of pancreatic cancers have a KRAS or BRAF mutation, supporting the notion that a KRAS mutation is not strictly required for the development of pancreatic cancer [22]. Of note, neither GNAS nor RNF43 mutations, which arose in cholangiocarcinoma as reported, were found in our samples. This suggests a low specificity for determining tissue origin based solely on single or multiple mutations. Therefore, here we propose a more effective omic approach.

**Table 1:** Summary of somatic mutations and coverage in the bile duct, omentum, and pancreas

	Mutation^a^	Gene	Range of somatic mutation	Coverage
Bile duct	84 (39)	52	0.01~0.333 (exon mutations);0.0437 ± 0.042 (non-exon mutations)	575.80 ± 338.69
Omentum	88 (41)	59	0.01~0.116 (exon mutations);0.043 ± 0.028 (non-exon mutations)	445.16 ± 230.39
Pancreas	63 (30)	40	0.011~0.127 (exon mutations);0.037 ± 0.024 (non-exon mutations)	399.14 ± 202.36

Notes: Mutations supported by either less than 100× coverage or fewer than 5 reads were not included. Only mutations that were not cataloged in dbSNP or found in the patient's blood DNA samples were included in the analysis.

^a^Total number of mutations found in each organ; exon mutations are shown in parentheses.

### Mutational concordance of the 390 key genes

**Figure 1:**
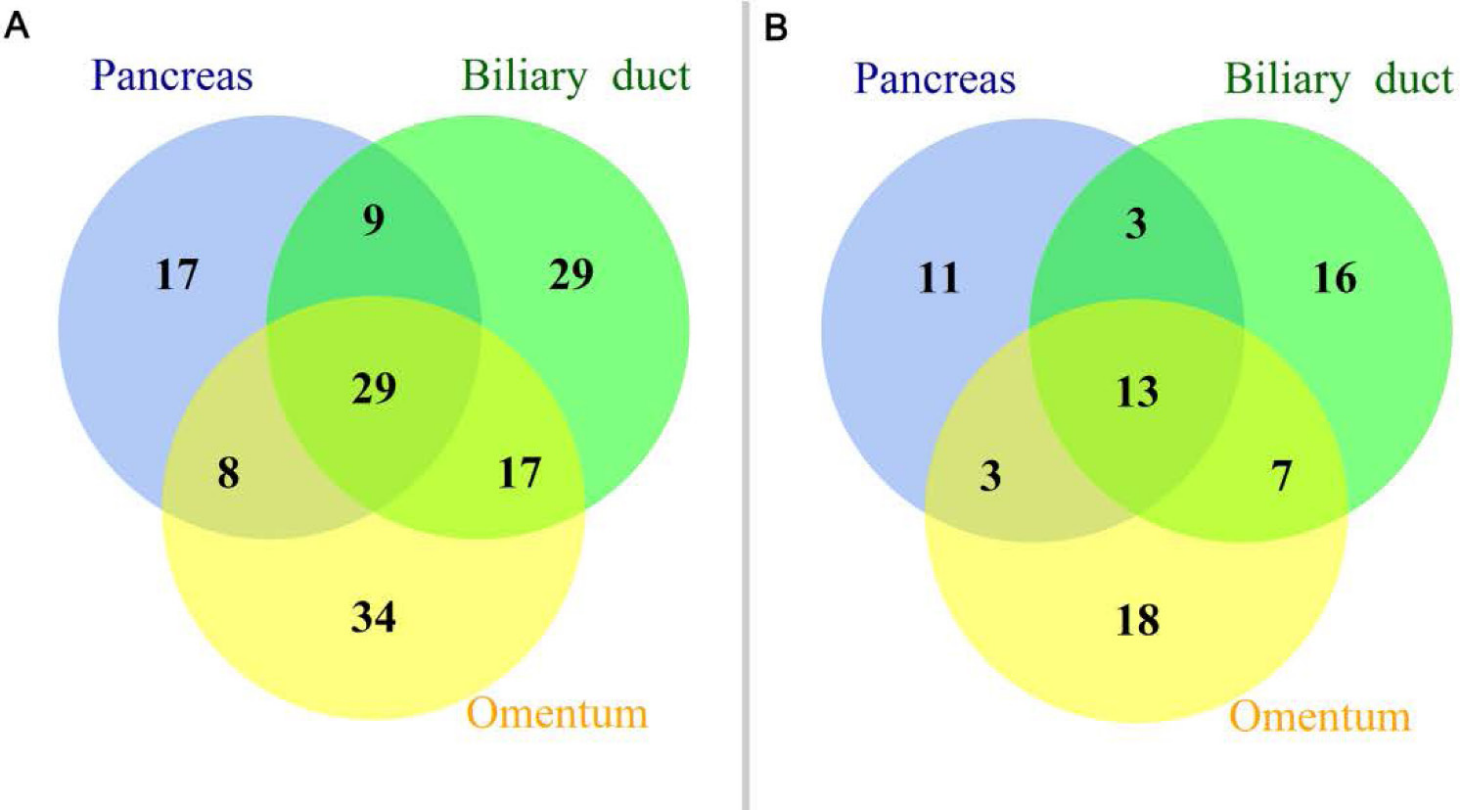
Mutational profiles of a panel of 390 key genes (**A**) Venn diagram depicting the shared somatic mutations detected in the tumors harvested from the biliary duct, omentum, and pancreas. (**B**) Venn diagram of somatic mutations in exons detected in the tumors harvested from the biliary duct, omentum, and pancreas.

**Figure 2:**
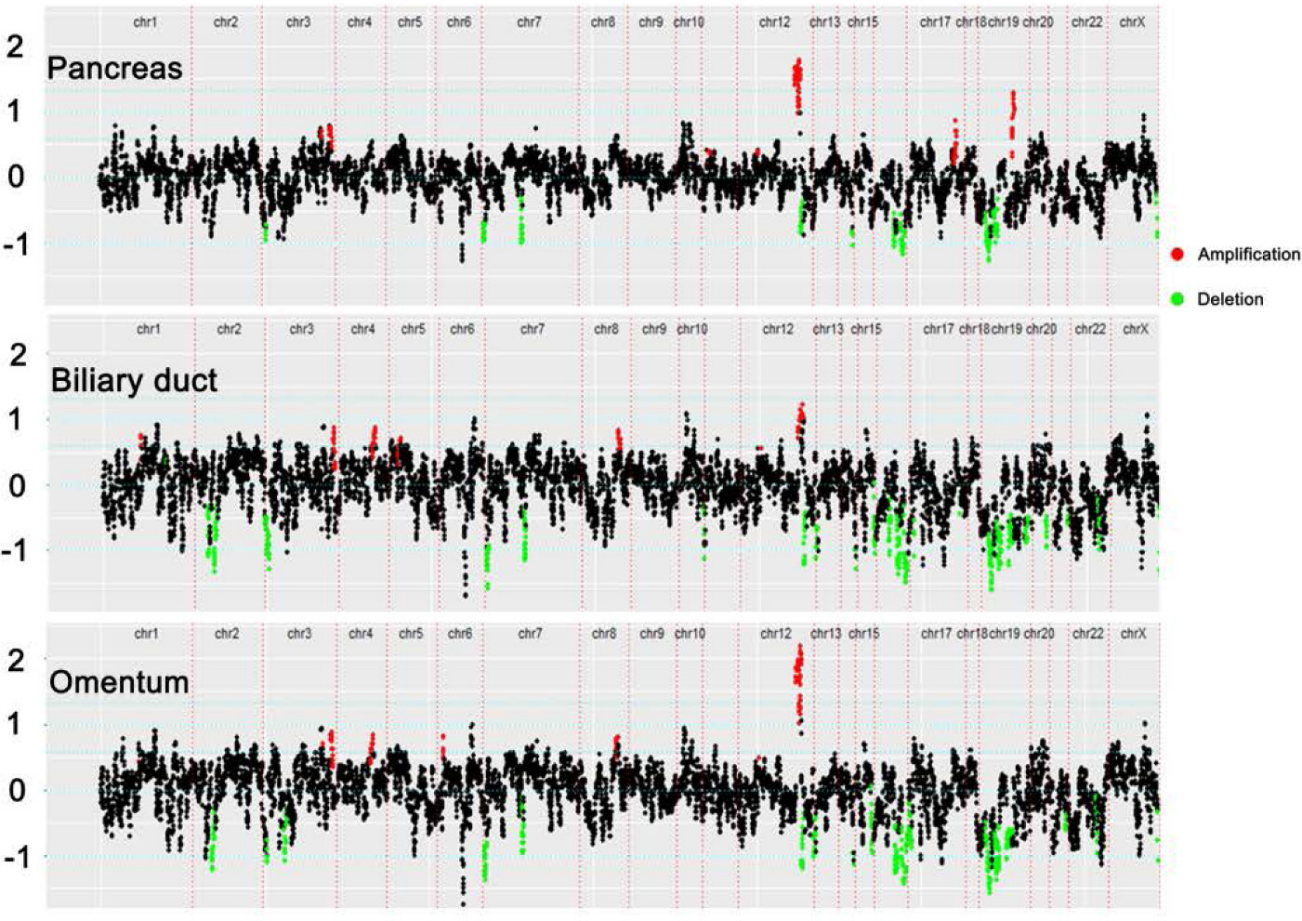
Copy-number variation (CNV) profiles of 390 key genes One dot on the plot refers to the read-depth ratio summarized by one probe; red indicates gene gain and green indicates gene loss.

According to the single clonal evolution theory of cancer cell populations [10], the observed somatic mutations or CNVs should harbor a certain amount of overlap if these three tumor deposits were of the same origin. In this study, we define somatic mutation as a shared mutation if it was detected inside the same gene and had the same genomic coordinate. Figure 1 shows that 38, 37, and 46 somatic mutations were shared between the pancreas and biliary duct, pancreas and omentum, and biliary duct and omentum, respectively. Concordant mutations between paired cancers were popular, especially between the biliary duct and omentum (> 50%). Twenty-nine somatic mutations were shared across these three tumors, and 13 of them were inside exons. As the probability of the occurrence of the same mutation at two genetically independent tumors is rare, especially for selected key cancer-related genes, the Venn diagram in Figure 1 suggests a single clonal origin for the observed cancer cell populations. The CNV profiles of 390 key genes (Figure 2) determined by NGS and found in these three samples were highly similar. We examined the overlap of CNVs across these 390 genes. All five CNVs (MDM2 gain, VHL loss, PLA2G1B loss, LIMK1 loss, and KEAP1 loss) found in the pancreas were shared in the other two samples. For the biliary duct and omentum, additional CDK4 gain and GLI1 gain were also shared, consistent with the notion that they were likely derived from the same clone.

### Whole-genome CNVs analysis

Whole-genome CNVs were analyzed across these three samples (Supplementary Figure S2). There was a high log2 profile similarity between the bile duct and the omentum, particularly on chromosomes 3, 8, and 12. This might reflect the close relationship between the composition of the tumor masses in the biliary duct and omentum, consistent with that observed in the NGS analysis above. In total, 185, 145, and 84 CNVs were detected in the bile duct, omentum, and pancreas across 22 autosomes and the sex chromosome (Supplementary Figure S3). The detailed CNV data for these three masses is listed in Supplementary Table S2.

We also examined the overlap of CNVs at the chromosome level among the tumor lesions. Only CNVs with identical intervals were defined as overlapping. Between the biliary duct and omentum, 6 CNV events were shared on chromosome 4 (143,211,964–146,188,881bp), chromosome 12 (57,810,254–58,498,926bp), chromosome 13 (48,964,265–49,017,901bp), chromosome 13 (86,031,023–90,196,888bp), chromosome 16 (30,095,734–35,271,725bp), and also on the Y chromosome (2,660,163–28,799,935bp); between the biliary duct and the pancreas, one CNV was shared on the X chromosome (177,942–2,686,899bp). However, no overlap was detected between the pancreas and the omentum. These results also led to the conclusion that the tumor masses in the biliary duct and the omentum were very closely related, but relatively far from that in the pancreas.

**Figure 3:**
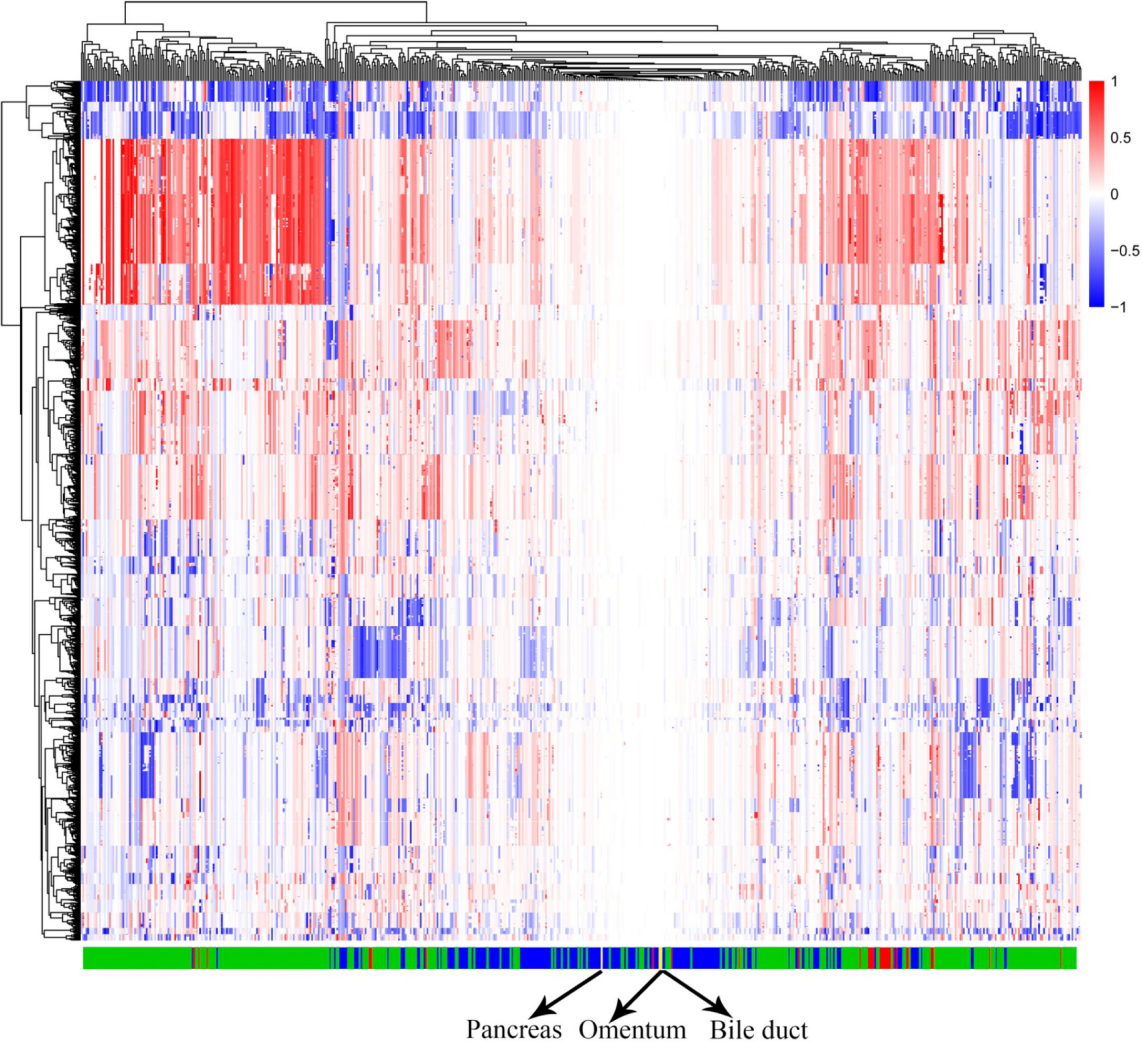
CNV model of three cancer types of the hepatobiliary and pancreatic system Columns: characteristic CNV regions based on CNTools analysis across all cancer samples. Rows: samples from The Cancer Genome Atlas (TCGA) and the patient; red: cholangiocarcinoma samples; green: hepatocellular carcinoma samples; blue: pancreatic carcinoma samples; yellow: three samples from the patient. As shown in the figure, most of the samples belonged to one cancer type, as they clustered together. The three samples from the patient were included as a type of pancreatic carcinoma.

By modeling the whole-genome CNV data from the TCGA, we calculated the accuracy of the CNV signature-generating computational model for each cancer. The results showed that > 84% of cholangiocarcinoma, > 88% of hepatocellular carcinoma, and > 99% of pancreatic cancer could be classified into their corresponding categories based on the selected CNV signatures. We then added the patient's whole-genome CNV chip data to the model obtained in the discriminant analysis above, and conducted the classification again. This analysis classified all three of the patient's samples as a type of pancreatic cancer, indicating that the patient likely had pancreatic cancer that metastasized to the biliary duct and omentum (Figure 3).

## DISCUSSION

The patient with cancer in this study had synchronous multifocal tumors in the pancreatic tail, upper biliary duct, and omentum at the time of diagnosis; although this condition is rare, it exemplifies a diagnostic challenge in the clinic. The treatment for patients may differ according to the cancer origin. If the initial patient diagnosis is pancreatic cancer with multiple metastases, surgery can be avoided and a more suitable systemic treatment could be initiated. Previous analyses of cytogenetic or chromosome inactivation data [23, 24] as well as specific analyses of genetic loci [25, 26] have been used to infer the origin of cancers; recent developments of genetic analyses of cancer-related gene panels or whole-genome analysis have also been applied [16, 27]. Often, the tissue origin of synchronous multifocal tumors is clear and analysis is then employed to assess whether they came from the same clone. However, in this study, we analyzed the relationship between synchronous multifocal tumors of an unknown origin. In this study, we used multi-omics analysis to infer the clonality of multifocal tumors across multiple tissues. In this patient, both the NGS data from a panel of 390 key genes and the CNVs data from a whole-genome chip revealed that the cancers found in the patient's pancreas, biliary duct, and omentum were clonally related at the genomic level. These findings are consistent with the single clonal evolution theory of cancer [10, 13].

The process of metastasis itself may represent an evolutionary event [28]. We consequently inferred the metastatic process of this cancer patient using evolutionary information revealed in the cancer cell populations. Both the somatic mutation data and CNVs revealed that the cancer cell populations sampled from the biliary duct and omentum were more related to each other than to those detected in the pancreas. Particularly for whole-genome CNVs, the overlap in the log2 scale indicated that the populations resembled each other, indicating a reasonably close relationship of cancer cell populations between the biliary duct and omentum, while such a relationship between the omentum and the pancreas was weak. Therefore, the tumor cells sampled from the omentum likely migrated from the biliary duct as opposed to those found in the pancreas.

Between biliary duct and pancreas tumors, the pancreas was more likely the origin of the cancer cells. When cancer cells invade a distant organ, they often gain additional biological behaviors and acquire additional genetic alterations. Both in somatic mutations and CNVs (whether in the 390 key genes tested or at the chromosomal level), we observed a greater number of biological events in cancer cells from the biliary duct. Mutational processes evolve throughout a cancer's lifespan, with many emerging late but contributing genetic variations. As subclonal diversification was prominent, we divided the genetic variations shared by all cancer cells originating from the same clone and those subclonal variants that occurred after the emergence of the common ancestor [29]. Considering the genetic heterogeneity of tumors, determining their clonal origin using individual coincidence of somatic genetic aberrations only is biased. Therefore, we built a computational model and clustered the three analyzed masses into the pancreatic cancer branch. Compared with data from 390 key genes, comprehensive whole-genome data seemed to be more suited to determine the tissue origin of multiple monoclonal tumors across multiple tissues, consistent with previous findings [18, 30].

We also investigated the time of metastasis in the examined patient. It reportedly takes 0.5~13 years (95% confidence interval) for a parental cancer clone in the pancreas to develop metastatic capacity [19]. Given the relatively distinct mutation signatures between the biliary duct and the pancreas, it seems that cancer cells in the pancreas migrated into the bile duct at a relative early stage according to the moderate number of shared mutations and CNVs. The cancer cells in the biliary duct invaded the omentum relatively late, as suggested by the highly similar CNV profiles between the bile duct and omentum.

In conclusion, our findings suggest that genomic analyse of cancer-relevant genes suffices to characterize the clonality between tumors when the tissue origin of the tumors is clear. However, for synchronous multifocal tumors of unknown origin across multiple tissues, more comprehensive whole genomic analysis should be applied. When there are no effective immunohistochemical markers to determine the origin of cancer cells, as for the patient we studied, genomic analyses could facilitate precision medicine. The method we have developed could also be extended to other solid tumors and might facilitate the accurate diagnosis of cancers with unknown primary origins.

## MATERIALS AND METHODS

### Clinical history

**Figure 4:**
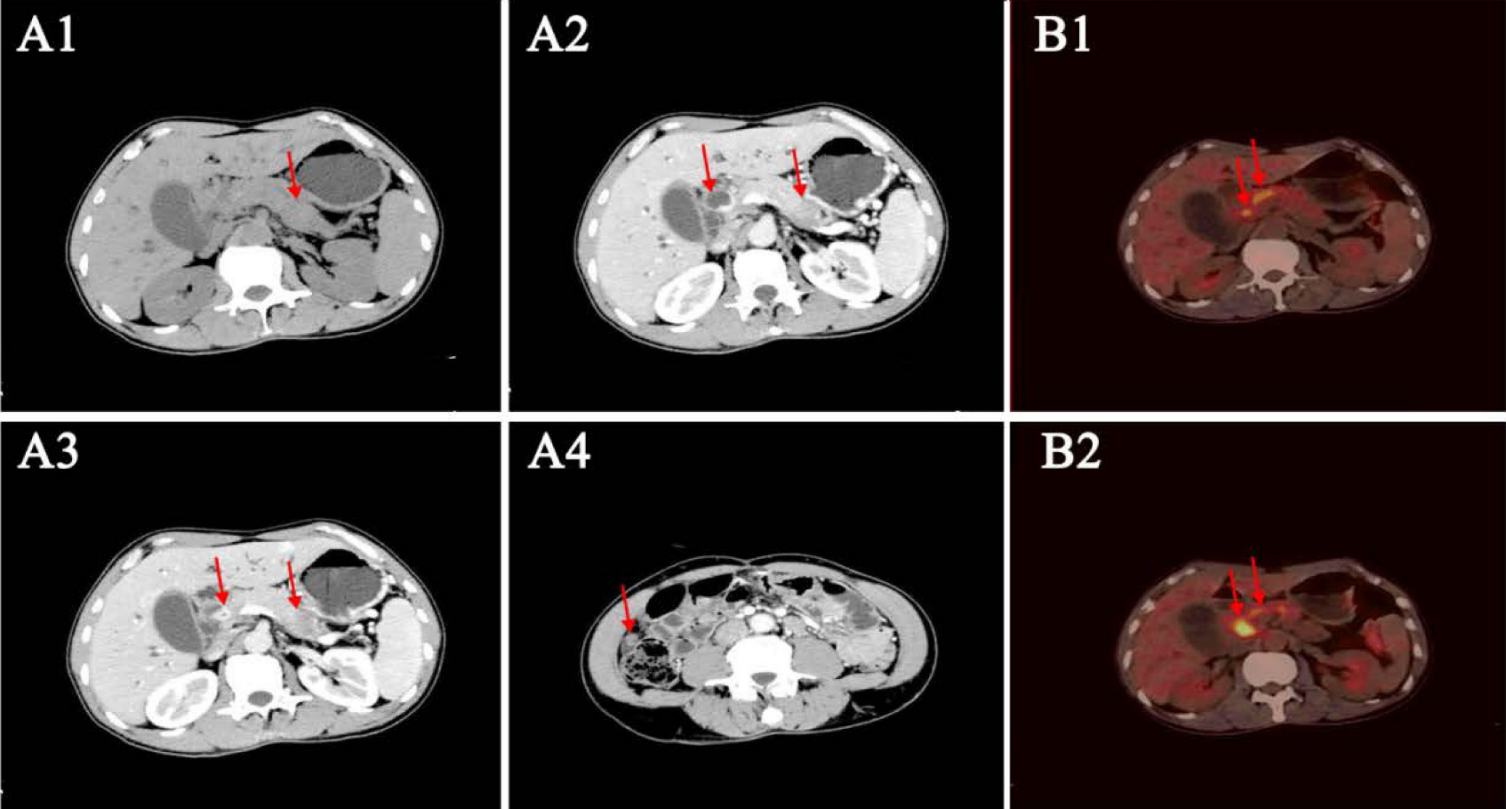
Radiological assessments of the patient (**A1**) Unenhanced computed tomography (CT) depicts an equal-density mass in the tail of the pancreas; the common bile duct is not clear. (**A2**–**A3**). Contrast-enhanced CT shows thickening of the wall of the common bile duct with significantly persistent enhancement. The mass in the pancreas tail is of low density compared with the dramatically enhanced mass in the pancreas. (**A4**). Contrast-enhanced CT shows mild enhancement of a mass in the right peritoneum. (**B1**–**B2**). Positron emission tomography (PET)/CT assessment of the patient. The PET/CT shows hypermetabolic masses in the comment bile duct and the head of the pancreas.

In 2015, a 50-year-old Chinese male with a history of ~6-months of recurrent abdominal pain and jaundice was referred to the First Affiliated Hospital of Zhejiang University, Hangzhou, Zhejiang Province, China. Computerized tomography (CT) revealed three separate masses in his pancreatic tail, upper common bile duct, and omentum. This pattern represents a typical imaging finding of pancreatic carcinoma with concomitant cholangiocarcinoma, suggesting the possibility of synchronous multiple primary tumors (Figure 4A). A positron emission tomography (PET)/CT scan was subsequently performed and showed hypermetabolic masses in the common bile duct and pancreas but not the omentum (Figure 4B). A complete blood count, chemistry profile, and tumor markers were obtained, including carbohydrate antigen 199 (CA-199) (486 U/mL; upper limit of 37 U/mL) and total bilirubin (TB)(68 U/mL; upper limit of 21 U/mL), indicating that the patient's clinical symptoms, laboratory tests, and imaging findings were consistent.

**Figure 5:**
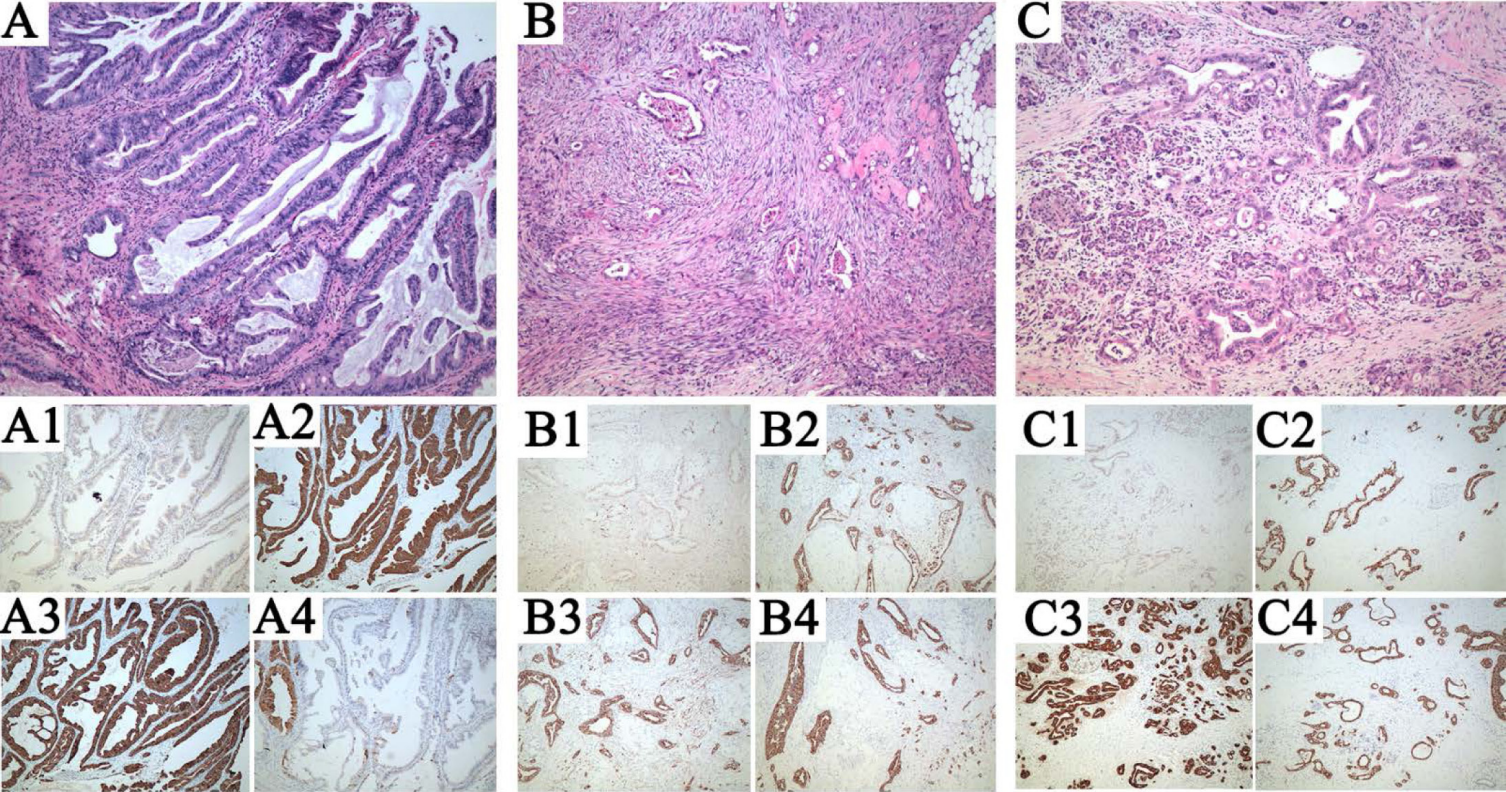
Pathological assessments of the patient (**A**) Representative micrographs of the upper biliary duct, (**B**) omentum, (**C**) pancreatic tail. (**A1**–**A4**), (**B1**–**B4**), (**C1**–**C4**). Caudal Type Homeobox (CDX)2, Cytokeratin (CK)7, CK19, CK20 immunohistochemical analysis of the tumors. Note that all lesions are composed of solid masses of atypical cells, with a high nuclear-cytoplasmic ratio and pleomorphic nuclei. CDX2 is negative in all lesions, meanwhile, CK7 and CK19 is expressed in all lesions. CK20 is positive in both pancreas tail and omentum, and partial positive in biliary duct. Hematoxylin & eosin staining; original magnification100×.

The patient had no prior personal history of cancer. An investigation of his family history did not find a confirmed case of cancer in his first-degree relatives. After a clinical diagnosis of synchronous pancreatic carcinoma and cholangiocarcinoma, a radical tumor resection was performed. Subsequent synchronous multiple malignant tumors were found in the upper biliary duct, and omentum, pancreatic tail. Histopathological analysis of these lesions revealed moderately differentiated adenocarcinomas (Figure 5A–5C). No metastasis was found in the regional lymph node dissection. Immunohistochemical analysis of these three masses demonstrated that both the omentum and the pancreatic tail were middle grade invasive ductal carcinoma positive for cytokeratin 7 (CK7), cytokeratin 19(CK19), cytokeratin 20(CK20) (Figure 2, 3, 4, 54, 5C2–5C4), but lacked Caudal Type Homeobox 2 (CDX2) expression (Figure 5B1, 5C1). The upper biliary duct mass was positive for cytokeratin 7 (CK7) (Figure 5A2), cytokeratin 19(CK19) (Figure 5A3), and partly positive for cytokeratin 20(CK20) (Figure 5A4), but lacked CDX2 expression (Figure 5A1).

Five months later, due to progressive weight loss and obvious fatigue, the patient visited our hospital again. Magnetic resonance imaging (MRI) showed multiple liver masses, with a higher level of CA-199 (644 U/mL; upper limit of 37 U/mL), indicating tumor metastasis. Based on the clinical history, we questioned the initial diagnosis of the patient. The histological and immunohistochemical findings were inconclusive as to whether these three masses found during surgery originated from the same clone or constituted synchronous multiple primary tumors. If the patient suffered synchronous pancreatic cancer and cholangiocarcinoma, which tissue the omental mass and subsequent liver masses originated from? Given these questions, we applied genomic analyses to the surgical samples and tried to infer the tumorigenesis of the patient.

Written informed consent was obtained from the patient for genomic examination and analyses of the samples. The internal review board of The First Affiliated Hospital, Zhejiang University approved the genetic analysis of the patient.

### DNA extraction and genomic analysis

For this patient, the representative histological cancer sections from the pancreas, bile duct, and omentum were reviewed and evaluated by a histopathologist. The proportion of cancer cells used from each tumor mass was at least 30% to maximize tumor cell content. DNA samples were extracted from paraffin-embedded tissues.

Next-generation sequencing (NGS) was performed using the targeted capture massively parallel sequencing platform (Illumina, San Diego, CA, USA). In order to detect somatic mutations, DNA extracted from peripheral blood was used as a control. A total of 390 key cancer-relevant genes were deep sequenced for potential mutations, which were called only on single base substitutions or small indel substitutions. To avoid potential sequencing errors, allelic mutations supported by either less than 100× coverage or fewer than five reads were not included. Only mutations that were neither cataloged in dbSNP nor found in the patient's blood DNA samples were included in the analysis; human genome build 19 was used as the reference for reported somatic mutations. We used CONTRA [31] and an in-house modified version of BIC-seq [32] to call CNVs following NGS. Genes that intersected segments with a read depth log2 ratio greater than 0.7 or smaller than −0.7 were selected as CNV candidates and were manually reviewed for precision.

In addition, whole-genome CNVs in the pancreas, bile duct, omentum, and blood were detected using an OncoScan^®^ CNV FFPE Assay Kit (Affymetrix, Santa Clara, CA, USA). The probes were used to capture the alleles of over 220,000 single nucleotide polymorphisms (SNPs) at carefully selected genomic locations, both those evenly distributed across the genome and those with increased density, within nearly 900 cancer-relevant genes. The CNV information was extracted from log2 track information and B-allele frequencies. The TuScan algorithm, an improved version of ASCAT [33], was used to determine the CNV events for the sampled tumor masses.

### Statistical and bioinformatics analyses

To determine whether the cell populations in the three samples came from the same clone, we first compared the similarities of 390 key gene somatic aberrations, including mutations and gene CNVs, between paired samples. For cancers of known origin, the majority of somatic aberrations in the primary tumors are shared with the corresponding metastatic lesions [19, 34]. For genetically independent cancers, the concordant gene aberrations were rare in the genomic landscape according to whole-exome sequencing [35].

TCGA CNVs from 595 cancer samples of hepatocellular carcinoma, pancreatic carcinoma, and cholangiocarcinoma were extracted and used to build a multi-cancer CNV dataset. We used the “CNTools” package in Bioconductor [36] to align the segmented DNA copy number data, and a total of 119,313 CNV regions were obtained. We then used generalized linear regression (‘glm’ in R) to select the CNV signatures, which were highly correlated with the cancer type. With a *p*-value < 1e-12 used as the cutoff, 12,127 cancer-specific CNV signatures were found for different cancer types. We then conducted a discriminant analysis [37] using SAS software [38] to further test how well the samples of these three cancer types could be classified into the corresponding categories based on the selected CNVs signatures.

## SUPPLEMENTARY MATERIALS FIGURES AND TABLES






