# Supplementary material for: Identifying the clonal origin of synchronous multifocal tumors in the hepatobiliary and pancreatic system using multi-omic platforms
Source: Oncotarget. 2016 Dec 19;8(3):5016–25. doi: 10.18632/oncotarget.14018 (PMC5354888; doi:10.18632/oncotarget.14018)
Supplement: Supplementary file 1 [file oncotarget-08-5016-s001.pdf]

# Identifying the clonal origin of synchronous multifocal tumors in the hepatobiliary and pancreatic system using multi-omic platforms

## Supplementary Materials

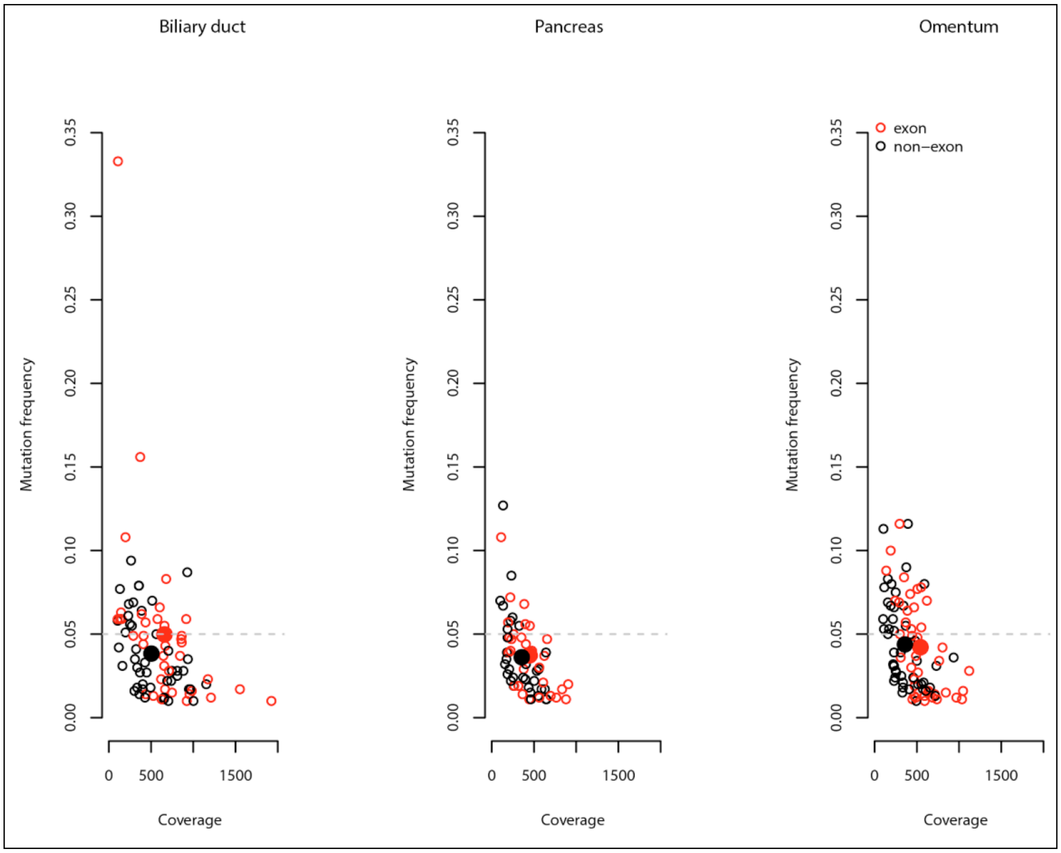


**Supplementary Figure S1: Coverage and frequencies of somatic mutations across three tumor masses.** Only the somatic mutations that had at least 100× coverage or more than 5 reads were included. The x-axis represents the coverage sequenced; the y-axis represents the somatic mutation rate.

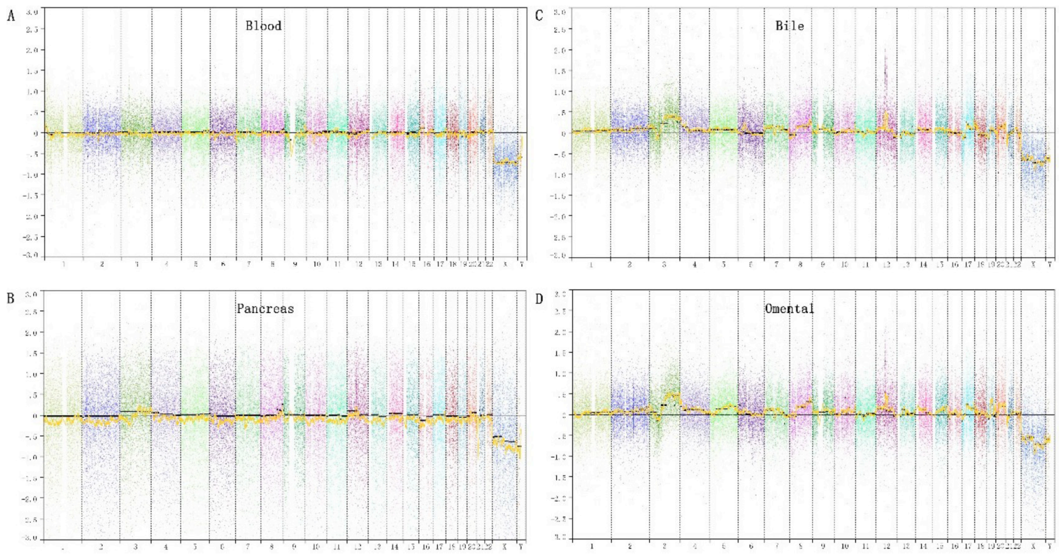


**Supplementary Figure S2: Genomic copy-number variation (CNV) profiles of the pancreas, biliary duct, and omentum.** This figure shows the log2 profiles of the genomes, demonstrating the effects of non-aberrant cell involvement and tumor cell aneuploidy.

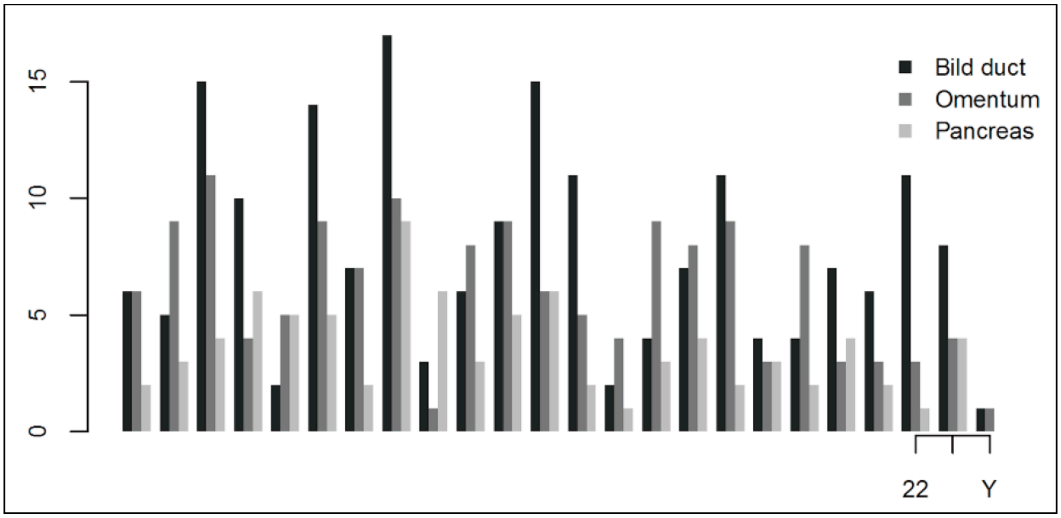


**Supplementary Figure S3: Distribution of CNVs across the genome for three samples from the patient.** In total, there were 184, 145, and 84 CNV events detected in the bile duct, omentum, and pancreas, respectively.

**Supplementary Table S1: Mutation events data of 390 key cancer-relevant genes across three tumor masses.** See Supplementary_Table_S1

**Supplementary Table S2: Whole-genome copy number variation (CNV) events data across three tumor masses.** See Supplementary_Table_S2
